# Cystic fibrosis testing in a referral laboratory: results and lessons from a six-year period

**DOI:** 10.1186/2043-9113-3-3

**Published:** 2013-01-23

**Authors:** Perry G Ridge, Christine Miller, Pinar Bayrak-Toydemir, D Hunter Best, Rong Mao, Jeffrey J Swensen, Elaine Lyon, Karl V Voelkerding

**Affiliations:** 1 ARUP Institute for Clinical and Experimental Pathology, Salt Lake City, UT, USA; 2 Department of Biology, Brigham Young University, Provo, UT, USA; 3 ARUP Laboratories, Salt Lake City, UT, USA; 4 Department of Pathology, University of Utah School of Medicine, Salt Lake City, UT, USA; 5 500 W Chipeta Way, Salt Lake City, UT, 84108, USA

**Keywords:** Cystic fibrosis, CFTR, Novel variants, Next-generation sequencing, Interpretation of variants

## Abstract

**Background:**

The recent introduction of high throughput sequencing technologies into clinical genetics has made it practical to simultaneously sequence many genes. In contrast, previous technologies limited sequencing based tests to only a handful of genes. While the ability to more accurately diagnose inherited diseases is a great benefit it introduces specific challenges. Interpretation of missense mutations continues to be challenging and the number of variants of uncertain significance continues to grow.

**Results:**

We leveraged the data available at ARUP Laboratories, a major reference laboratory, for the *CFTR* gene to explore specific challenges related to variant interpretation, including a focus on understanding ethnic-specific variants and an evaluation of existing databases for clinical interpretation of variants. In this study we analyzed 555 patients representing eight different ethnic groups. We observed 184 different variants, most of which were ethnic group specific. Eighty-five percent of these variants were present in the Cystic Fibrosis Mutation Database, whereas the Human Mutation Database and dbSNP/1000 Genomes had far fewer of the observed variants. Finally, 21 of the variants were novel and we report these variants and their clinical classifications.

**Conclusions:**

Based on our analyses of data from six years of *CFTR* testing at ARUP Laboratories a more comprehensive, clinical grade database is needed for the accurate interpretation of observed variants. Furthermore, there is a particular need for more and better information regarding variants from individuals of non-Caucasian ethnicity.

## Background

Over the past few years, large scale sequencing efforts have provided a greater understanding of the variability of the human genome. Notably, whole genome sequencing studies have shown that each individual harbors 2.7–4.2 million single nucleotide variants (SNVs) that differ from the human reference genome [1], whereas exome sequencing typically identifies 18–24,000 coding region based SNVs per individual [2-7]. With regard to SNVs in coding regions, the findings generated by whole genome or exome sequencing parallel those observed when sequencing individual genes. First, a majority of identified variants are present in dbSNP [8,9] and therefore represent more common variation. For example, exome and genome sequencing reports have shown that 88–99% of observed SNVs reside in dbSNP [1-3,5,10]. Second, the number of SNVs is dependent on individual genetic variability, ethnicity, and the reference sequence to which results are aligned and compared. At present, most studies utilize the human genome reference sequence for alignment (hg18/GRCh36 or hg19/GRCh37), which shares greatest similarity to Caucasian individuals of Northern European ancestry. As a consequence, the number of SNVs observed can vary considerably depending on the ethnic background of samples. Third, at the individual gene level, variants may have already been described and classified in a gene specific database. Not infrequently, however, novel SNVs are identified, even in genes that have been extensively studied through clinical research or diagnostic testing. While guidelines exist to assist in SNV annotation and functional prediction [11-14], many novel variants continue to be classified as variants of uncertain significance (VUS). As a greater number of exomes and genomes are sequenced, having a more comprehensive catalogue of human genetic variation will facilitate individual gene variant classification.

In the context of the above observations, we proposed that analysis of a dataset of variants identified in a single gene would yield insights into what will be revealed by large scale sequencing studies going forward. In our referral laboratory setting, we chose to study the cystic fibrosis transmembrane conductance regulator (*CFTR*) gene, representing a high volume full gene sequencing diagnostic assay. It should be noted, however, that at ARUP Laboratories, most cases have previously undergone testing with a 32-mutation panel identifying the most common disease-causing alleles, before sequencing. Thus, sequencing results are enriched for rare *CFTR* mutations. *CFTR* (NM_000492) is located at 7q31.2 and consists of 27 exons coding for a 1480 amino acid protein, which is a member of the ATP-binding cassette (ABC) transporter superfamily. Mutations in *CFTR* are known to result in multiple conditions, ranging from classic cystic fibrosis (CF) to monosymptomatic diseases such as congenital absence of the vas deferens, pancreatitis, or chronic bronchiectasis.

Classic CF, a recessively inherited genetic disorder, has an incidence of one in 2500–3200 in Caucasians making it one of the most common lethal genetic disorders [15]. CF occurs with different frequencies in different ethnic groups with estimated carrier rates of one in 28, 29, 46, 65, and 90 in Caucasians, Ashkenazi Jews, Hispanics, African Americans, and Asians, respectively [16,17]. The American College of Medical Genetics recommends carrier screening for CF in expectant individuals or those planning a pregnancy by testing for 23 known disease-causing mutations [18]; between 48% and 84% of clinically diagnosed CF patients have at least one of these mutations [19]. The most common *CFTR* gene mutation is a three base pair deletion, p.Phe508del (prevalence of 24%-88% depending on ethnic background [17,19-21]), which is associated with a more severe phenotype when present in a homozygous state [21]. Similarly, other variants have variable frequency in different populations [22]. In all ethnic groups, the majority of *CFTR* variants are of unknown clinical significance [22,23]. Several databases have reported variants in *CFTR* including dbSNP [8,9], the Cystic Fibrosis Mutation Database (CFMDB) [24], and the Human Gene Mutation Database (HGMD) [25]. Variants in HGMD are assumed to be disease causing, but there are exceptions. Variants in dbSNP, on the other hand, are often assumed to be benign; however, that is not always the case. The CFMDB contains both disease causing and benign variants.

Herein we present results from a six-year period of *CFTR* diagnostic testing, including 21 novel variants, during which samples from 1407 individuals were referred to ARUP Laboratories for full gene *CFTR* sequencing. We focus on the need to develop a more complete understanding of variants in non-Caucasian ethnic groups, evaluate the usefulness and completeness of databases for clinical testing, and report novel variants observed at ARUP with ethnicity and clinical classifications.

## Methods

### Description of dataset

The dataset for the current study was comprised of variants identified through *CFTR* gene sequencing from 555 patients referred to the ARUP Laboratories from 2004 to March of 2011. Indications for testing included carrier testing in healthy individuals, confirmation or diagnostic testing in patient with classically affected CF, and diagnostic testing for patients with potentially CF-related symptoms, but without a diagnosis of CF. Patients self-reported ethnicity from the following categories: African American, Ashkenazi Jewish, Asian/Oriental, Caucasian, Hispanic, Mediterranean, Mid-Eastern, Native American, Other, or any combination of the above categories. All patient information (including demographic information, clinical symptoms, and laboratory reports including sequencing results) was stored in a Progeny database [26].

Samples were sequenced bi-directionally by a Sanger method using dye-terminator chemistry (BigDye^®^ Direct Cycle Sequencing kit; Life Technologies, Carlsbad, CA) with M13-tailed primers. Sequencing products were separated and detected by capillary electrophoresis (ABI 3730; Life Technologies). All 27 exons of the CFTR gene and intron/exon boundaries were interrogated. The sequence was analyzed with Mutation Surveyor^®^ (SoftGenetics, State College, PA) by two independent reviewers and a third and final review by a board certified (ABMG) clinical molecular geneticist.

### Correlation with existing databases

Sorting and correlation of sequence changes between ethnic groups and with databases, and functional classifications of variants were completed using a series of custom-built Java programs. Databases queried included dbSNP build 132 [8,9], which includes variants from the 1000 Genomes Project [27], CFMDB [24], downloaded May 2011, and the professional version of HGMD [25] (as of May 2011).

### Variant classification

Within our institution, clinical molecular geneticists used the following steps to classify variants. First, relevant databases were interrogated to see if the sequence change had been previously observed, and if present, how it was reported. For this study, a local Progeny database, dbSNP, the Human Gene Mutation Database (HGMD), and the Cystic Fibrosis Mutation Database (CFMDB) were searched. Next, literature searches were performed to see if functional consequences had been previously reported for the variant. If the variant was not previously reported in the literature its frequency was determined by cross-referencing dbSNP/1000 Genomes data. In addition, *in silico* prediction algorithms (PolyPhen [28], SIFT [29], PSAAP [30], Human Splicing Finder [31], MaxEntScan [32], etc.) were used. While *in silico* prediction was employed in the overall evaluation of certain variants, no classification was based solely on *in silico* predictors. Lastly, unless evidence existed to the contrary, common, synonymous, and deep intronic (more than 20 base pairs into the intron) SNVs were typically reported as benign or suspected benign (depending on the frequency).

## Results

### Total and ethnic specific variants

To determine variant distribution within the *CFTR* gene and in separate ethnic groups, we analyzed observed variants from 555 patient referral samples for which ethnicity data was available and at least one single nucleotide variant (SNV) was identified. The nine self-reported ethnicities selectable by patients were Mediterranean, Ashkenazi Jewish, Native American, Mid-Eastern, Asian/Oriental, Hispanic, Other/Mixed, African American, and Caucasian (in order of increasing numbers of patients).

A total of 184 different SNVs were observed in our dataset, 107 of which were observed only a single time (if a patient were homozygous for the variant, this was designated as two observations) and 16 were observed 10 or more times. The four most common variants: c.1408A > G (p.M470V), c.2562 T > G (synonymous, p.Thr854Thr), c.4389 G > A (synonymous, p.Gln1463Gln), and c.869 + 11C > T (intronic) were observed 955, 728, 427, and 236 times, respectively, and each is classified as a common polymorphism.

Total and ethnic specific variants (i.e., variants found in only one ethnic group in this study) were calculated for each group (Table 1). Total SNVs ranged from one each in the Ashkenazi Jewish and Native American groups to 125 in the Caucasian group. No ethnic specific SNVs were observed in the Ashkenazi Jewish and Native American groups whereas 98 Caucasian specific variants were identified. With one exception, the percentage of variants which were ethnic specific increased with increasing numbers of patients, the one exception being the Middle Eastern group (3 patients) where 60% of identified SNVs were ethnic specific. This is the second highest percentage following the Caucasian group with 78% ethnic specific SNVs.

**Table 1:** **Shown are numbers of patients with variants, total SNVs observed in a particular ethnic group, ethnic specific SNVs (where ethnic specific SNVs are SNVs seen in only one ethnic group in this study), and SNVs that were both novel (not present in any of the three databases in Table**2**) and ethnic specific**

**Ethnicity**	**# patients**	**SNVs**		
		**Total**	**Ethnic Specific**	**Novel & Ethnic Specific**
African American	61	36	19 (52.78%)	5 (13.89%)
Ashkenazi Jewish	1	1	0 (0%)	0 (0%)
Asian/Oriental	4	4	1 (25.00%)	1 (25.00%)
Caucasian	403	125	98 (78.40%)	11 (8.80%)
Hispanic	40	28	10 (35.71%)	2 (7.14%)
Middle Eastern	3	5	3 (60.00%)	1 (20.00%)
Native American	1	1	0 (0%)	0 (0%)
Other/Mixed	42	26	N/A	1 (3.85%)

Shown are numbers of patients with variants, total SNVs observed in a particular ethnic group, ethnic specific SNVs (where ethnic specific SNVs are SNVs seen in only one ethnic group in this study), and SNVs that were both novel (not present in any of the three databases in Table 2) and ethnic specific. Ethnicities were self-reported and reported percentages were percentages of the total SNVs.

### Novel and database variants

To determine numbers of novel SNVs versus those present in existing databases, all identified SNVs were cross referenced with dbSNP (release 132 which included the 1000 Genomes Project variants [27]), the Cystic Fibrosis Mutation Database (CFMDB), and the Human Gene Mutation Database (HGMD), with results summarized in Table 2. At the time of accessioning for this study, the dbSNP, CFMDB and HGMD databases contained 1430, 1383 and 1057 *CFTR* SNVs, respectively. A total of 184 different SNVs were observed in our dataset, and 163 (89%) were present in one or more of the cross-referenced databases. In comparison, 21 (11%) of the 184 SNVs were not present in any of the three databases and were therefore classified as novel (Additional file 1 contains substantial clinical and interpretive information for each of the novel variants). With respect to previously observed SNVs, 156 were present in CFDMB compared to 118 and 74 in HGMD and dbSNP, respectively (Table 2).

**Table 2:** The number of SNVs found in each of three databases, as well as totals is reported

	**SNVs in database (%)**	**SNVs not in database (%)**
Cystic Fibrosis Mutation Database	156 (84.78%)	28 (15.22%)
Human Gene Mutation Database	118 (64.13%)	66 (35.87%)
dbSNP 132	74 (40.22%)	110 (59.78%)
**Total**	**163 (88.59%)**	**21 (11.41%)**

The number of SNVs found in each of three databases, as well as totals is reported. In total, 88.59% of SNVs were found in at least one of the three databases.

Each of the 21 novel variants was only observed a single time in our dataset. The novel variants were classified by type with 11 variants residing in exons (8 missense, 2 nonsense, and 1 synonymous), 9 residing in introns, and a single variant upstream of the translational start site. Because these variants were confirmed bi-directionally by Sanger sequencing, which has a specificity over 99% [33], these variants are considered true positives. Three variants, inclusive of those mentioned, were assigned to two classes (intronic and splice site, or missense and splice site). Novel variants were classified as described in Methods with 8 variants classified as benign, 3 as suspected benign, 4 as disease causing, and 6 of unknown significance.

### Functional annotation and variant significance

Variants, including novel SNVs, were divided into five different groups (with some SNVs placed into multiple groups): splice site (defined as SNVs in the four positions flanking exon/intron boundaries), promoter (any position upstream of the translational start site), intronic, nonsense, missense, or synonymous (Table 3). The majority of SNVs (57%) were missense, followed by synonymous (18%), intronic (17%), splice site (10%), nonsense (6%), and promoter (2%). Next, the 184 observed variants were divided into five classes based on classification: 97 pathogenic, 8 suspected pathogenic, 37 benign, 30 of unknown significance, 6 suspected benign, and 6 with no recorded significance in our local database.

**Table 3:** Variant classifications

**Variant class**	**Number of variants**
Missense	105
Intronic	32
Synonymous	33
Splice Site	18
Nonsense	11
Promoter	3
**Total**	**184**

Number of variants assigned to each of six possible classifications. Some variants were assigned to multiple classes (i.e. intronic and splice site). Promoter variants are variants upstream of the translational start site, and a splice site variant is a variant located in one of the four positions flanking exon/intron (and intron/exon) boundaries.

## Discussion

These data were derived from six years of *CFTR* gene sequence analysis at a national reference laboratory. While Sanger-based sequencing assays of a single gene or a few genes are contemporary practice, a shift is ongoing towards the development and implementation of larger gene panels performed by next generation sequencing. Furthermore, it is anticipated that exome and whole genome sequencing will also transition from a research to a diagnostic tool, with early examples already reported [34]. The current study results are relevant in the context of current single-gene Sanger-based assays and larger scale gene sequencing.

Based on published whole genome and exome studies showing high percentages of observed SNVs in dbSNP, we expected the majority of SNVs in the *CFTR* gene to be present in dbSNP. Surprisingly, only 40% of observed SNVs in our dataset were in dbSNP. This relatively low number of observed SNVs in dbSNP can be easily explained. Common variants are more likely to be present in dbSNP than rare variants, and our dataset is enriched for rare variation. This is because, in addition to full gene sequencing, ARUP also offers a common mutation panel for *CFTR*, and sequenced patients are typically those with symptoms of CF, whose common mutation panel did not identify two pathogenic mutations. dbSNP is an effective catalogue for common variation, but has limited information about private variants (estimated to be thousands per individual [35]) and is not considered a clinical grade database.

In contrast to dbSNP, the majority of SNVs were present in CFMDB, the locus specific database. This demonstrates two key points. First, in order to interpret SNVs it is important to have sequence information for a large number of individuals. In our dataset, even after six years and more than 1000 individuals, the majority of SNVs (107 of 184) were only observed a single time. Second, locus specific mutation databases are important resources for variant interpretation as they often feature a better representation of the rare variation present at a particular locus and they generally include phenotype information about the variants.

A challenge common to sequencing studies that range from analyzing a single locus to analyzing an entire genome is variant interpretation. The first step in any study is to determine the location of variants relative to a chosen reference sequence, and the choice of reference sequence has specific implications. For example, the reference sequence may contain minor alleles in certain positions, rather than the true wild type allele. Thus variants can be identified which are, in reality, representative of the more common alleles [1]. Next, it has been reported that most of the DNA used for sequencing of the NCBI reference genome came from a single, anonymous male donor (RPCI-11) from Buffalo, NY [36]. In our dataset, the majority of observed SNVs were specific to certain ethnicities. For interpretation, it is necessary to determine whether these represent normal polymorphic variants within the ethnic groups or rare pathogenic alleles. This is an important distinction to make since the functional significance of a SNV may depend on the genetic background of the individual, meaning that SNVs can have different consequences in different ethnic groups [37,38]. The importance of considering ethnic background was demonstrated in a study utilizing different sets of variant panels for CF testing [20]. In this study, panels of 70 and 86 CF causing mutations yielded a detection rate of 85% in Caucasians and 95% in Ashkenazi Jews; however, only 58% and 62% in Hispanics and African Americans, respectively. When Hispanic and African American specific mutations were included in the panel, detection rates increased to ~95% in both groups [20]. Additionally, by utilizing a unique reference sequence based on ethnic-specific allele frequencies, the error rate in identifying disease-associated variants can be substantially reduced [39].

## Conclusions

As a result of our analyses, we have identified a number of challenges involving variant interpretation, such as identification of novel variants, choice of reference sequence, and ethnic background of the individual. These challenges are likely to extend, in greater magnitude, to gene panels, exomes, and genomes. As the scale of genomic information increases, the need for highly curated, clinical grade gene databases, such as the new ClinVar database, http://www.ncbi.nlm.nih.gov/clinvar/, will be increasingly pressing to facilitate interpretation.

## Abbreviations

CF: Cystic fibrosis; SNVs: Single nucleotide variants; VUS: Variants of uncertain significance; CTFR: Cystic fibrosis transmembrane conductance regulator; CFMDB: Cystic Fibrosis Mutation Database; HGMD: Human Gene Mutation Database.

## Competing interests

The authors declare that they have no competing interests.

## Authors’ contributions

PGR and KVV conceived and designed the study. PGR mined and processed the data, and wrote the necessary programs for the analyses. PGR, KVV, and EL analyzed the data. CM, PBT, DHB, RM, JJS, and EL interpreted the observed variants. PGR drafted the manuscript. All authors provided critical feedback on the manuscript and read and approved the final manuscript.

## Supplementary Material

Additional file 1 Novel variants. Click here for file
