# Supplementary material for: Cystic fibrosis testing in a referral laboratory: results and lessons from a six-year period
Source: J Clin Bioinforma. 2013 Jan 23;3:3. doi: 10.1186/2043-9113-3-3 (PMC3563502; doi:10.1186/2043-9113-3-3)
Supplement: Additional file 1 — Novel variants. [file 2043-9113-3-3-S1.pdf]

Additional File 1. Novel variants.

Variant	Type/Protein Change	Classification
c.-24A>G	Promoter	Suspected Benign
c.53+2T>C	Splice/Intronic	Mutation
c.53+21G>A*	Intronic	Benign
c.54-8T>A*	Intronic	Benign
c.273+35C>T	Intronic	Benign
c.441C>G	p.His147Gln	VUS
c.579+40A>G	Intronic	Benign
c.1210G>C	p.Gly404Arg/Splice?	VUS
c.1301C>A	p.Ser434X	Severe Pathogenic
c.1400T>C†	p.Leu467Pro	Severe Pathogenic
c.1486T>G	p.Trp496Gly	VUS
c.1679+9C>G	Intronic	Suspected Benign
c.1742T>A	p.Leu581X	VUS
c.2708A>G	p.Tyr903Cys	VUS
c.2909-6T>C	Intronic	Benign
c.2988+40T>A	Intronic	Suspected Benign
c.3080T>G	p.Ile1027Ser	Benign
c.3718G>T†	p.Val1240Leu/Splice?	VUS
c.3723C>A	p.Gly1241Gly	Benign
c.3849G>C	p.Arg1283Ser	At least mild mutation
c.4243-20A>G*	Intronic	Benign

Computer Prediction	Ethnicity	Age at Testing
N/A	Hispanic	Unknown
Predicted to change splicing	African-American	Unknown
No predicted splicing changes	Asian/Oriental	48
No predicted splicing changes	Caucasian	47
No predicted splicing changes	Caucasian	Unknown
Tolerated, Possibly Damaging	Caucasian	6
No predicted splicing changes	Caucasian	Unknown
Deleterious, Benign, No predicted splicing changes	African-American/Caucasian	11
N/A	African-American	Unknown
Deleterious, Probably Damaging	Caucasian	5
Tolerated, Probably Damaging	Caucasian	17
No predicted splicing changes	Hispanic	6
N/A	Caucasian	1
Tolerated, Benign	Caucasian	44
No predicted splicing changes	Middle Eastern	9
No predicted splicing changes	African-American	<1
Deleterious, Benign	Caucasian	1
Tolerated, Possibly Damaging, Predicted to change splicing	Caucasian	56
N/A	Caucasian	1
Deleterious, Probably Damaging	African-American	25
No predicted splicing changes	African-American	<1

Other Known Deleterious Mutations
None
None
None
None
None
None
None
None
c.1585-1G>A
c.1521_1523del (p.Phe508del)
c.3909C>G (p.Asn1303Lys)
None
None
None
5T-12TG
c.350G>A (p.Arg117His)
None
c.1001G>T (p.R334L)
None
c.3197G>A (p.Arg1066His)
c.224G>T (p.Arg75Leu), c.2988+1G>A

Clinical Symptoms
Chronic cough, pseudomonas, staph pneumonia, and pancreatic insufficiency
Failure to thrive, malabsorption due to pancreatic insufficiency
Pancreatitis
Pancreatitis, sinusitis, asthma
Unknown
Recent weight loss, NF1 diagnosis, ADHD, delays, chronic ear infections
Pancreatitis
Sinusitis
Failure to thrive, chronic cough
Pneumonia, chronic cough, height is 25% and weight is 10%
Pneumonia, chronic cough, lung disease
Sinusitis, asthma
Sinusitis
No symptoms (spouse of known CF carrier)
Pneumonia, chronic cough, bronchiectasis, sinusitis
Elevated IRT
Rectal prolapse
Chronic cough, sinusitis
Jaundiced, elevated liver enzymes, FTT
Failure to thrive, chronic cough, bronchiectasis, sinusitis, asthma, sinus surgeries revealed necrosis and inflammation
Abnormal newborn screen

Sweat Chloride Test
65 (High)
Unknown
Unknown
Unknown
Normal
49 (Borderline), 46
None
54 (Borderline), 62
High
97 (High)
59 (Borderline)
Borderline
None
Unknown
Normal
Unknown
Unknown
Unknown
Unknown
85 (High)
Unknown

Additional File 1: Novel variants (SNVs not found in any of the three databases) are shown along with the protein change/type of change, classification, in silico functional prediction, ethnicity, age at testing, the results of deletion and duplication (Del/Dup) testing, other known pathogenic mutations, clinical symptoms, results from sweat chloride tests (if performed), and family history of CF. Sweat chloride values consistently above 60 mmol/L are indicative of classic CF, while borderline values (40-59 mmol/L) may reflect some CF-like symptoms, but without a diagnosis of CF. Variants were assigned to one (or more) of six classes. Promoter variants are variants upstream of the translational start site, and a splice site variant is a variant located in one of the four positions flanking exon/intron (and intron/exon) boundaries. If an individual reported belonging to multiple ethnic groups, each of the groups is listed, separated by a forward slash. Predictions were made using SIFT, Polyphen-2, and a composite prediction from Alamut (Interactive Biosoftware, 2012). SIFT returned deleterious or tolerated (Kumar, *et al.*, 2009), Polyphen-2 returned benign, possibly damaging, or probably damaging (Adzhubei, *et al.*, 2010), and Alamut showed a graphic displaying whether or not there were predicted splicing changes.

*Indicates Deletion/Duplication analysis was performed and was negative, otherwise it was not performed

†Indicates a family history of CF with the p.Leu467Pro mutation also seen in an affected relative.
